# Sex Inequalities in Medical Research: A Systematic Scoping Review of the Literature

**DOI:** 10.1089/whr.2021.0083

**Published:** 2022-01-31

**Authors:** Lea Merone, Komla Tsey, Darren Russell, Cate Nagle

**Affiliations:** ^1^ School of Health Sciences, James Cook University, Townsville, Queensland, Australia.; ^2^ Cairns Sexual Health Service, Cairns North, Queensland, Australia.

**Keywords:** feminism, gender, health disparities, gender gaps, medicine

## Abstract

***Background:*** Historically, medical studies have excluded female participants and research data have been collected from males and generalized to females. The gender gap in medical research, alongside overarching misogyny, results in real-life disadvantages for female patients. This systematic scoping review of the literature aims to determine the extent of research into the medical research sex and gender gap and to assess the extent of misogyny, if any, in modern medical research.

***Methods:*** Initial literature searches were conducted using PubMed, Science Direct, PsychINFO and Google Scholar. Articles published between January 01, 2009, and December 31, 2019, were included. An article was deemed to display misogyny if it discussed the female aesthetic in terms of health, but did not measure health or could not be utilized to improve clinical practice.

***Results:*** Of the 17 included articles, 12 examined the gender gap in medical research and 5 demonstrated misogyny, assessing female attractiveness for alleged medical reasons. Females remain broadly under-represented in the medical literature, sex and gender are poorly reported and inadequately analyzed in research, and misogynistic perceptions continue to permeate the narrative.

***Conclusion:*** The gender gap and misogynistic studies remain present in the contemporary medical literature. Reasons and implications for practice are discussed.

## Background

Amid calls for Australian research policy to align with those in Europe and United States and increase equality in sex and gender recruitment in medical research,^1^ the sex and gender gap in medical practice is drawing increasing media attention.^2,3^ Females account for >50% of the global population and, therefore, a significant proportion of the patient population,^4^ yet women wait longer than men for both a diagnosis^5^ and pain relief, and^6,7^ are more likely to be misdiagnosed or discharged during serious medical events.^5^

Historically, medical studies have excluded female participants and research data have been collected from males and subsequently generalized to females^8^ and those who are intersex and do not have the reproductive anatomy characteristic of female or male.^9^ There are several postulated reasons for preferring males in research, including concerns for decreasing fertility or harming pregnancy,^10^ researcher bias from predominantly male researchers,^11^ and perception of the male as representative of the human species and, therefore, the norm.^12^

The gender gap in medical research, alongside overarching misogyny, results in real-life implications for female patients.^13^ The “Yentl syndrome,” named after the central protagonist in the 19th century story by Isaac Beshevis Singer, is the phrase coined by Dr. Bernadette Healy that describes how, for a woman's illness to be taken seriously, she must prove herself to be as unwell as a male counterpart.^14^ Yentl syndrome as a cause of delayed care for female patients is well documented in the medical literature.^13^ Lack of research evidence from female patients may result in delay in treatment; applying inappropriate, ineffective, or harmful treatments; or the withholding of effective treatments.

The resulting detriment to women's health is evidenced in the contemporary literature; recent studies have demonstrated that women with acute myocardial infarction (MI) present with different symptoms to men and are less likely to be identified during angiography than men, simply because they are often underinvestigated and subsequently less likely to be managed appropriately.^15^ Six-months after admission for MI, female patients, especially younger women, were more likely to suffer major adverse events and overall mortality.^15,16^

Androcentricity in medical research has historically disadvantaged and damaged female patients, from inaccurate diagnoses of “hysteria” and related barbaric treatments such as clitoridectomies and extended periods of enforced bedrest, to the more modern discrepancies observed in diagnoses and management of female patients.^17^ The hysterical discourse is often used colloquially, with terms such as “mad” and “crazy” used to describe “difficult” women who do not respond to treatment or diagnosis as expected.^18^

Young et al. examined the discourse surrounding patients with endometriosis, determining that the pain and experiences of these patients were often dismissed as psychological.^18^ In keeping with this, a recent study in the United States determined an average time to diagnosis of endometriosis from first consultation was 4.4 years and this was shorter in women who were aged <18 years and 40–49 years and those referred to and investigated by gynecologists.^19^

The concept of females being “difficult” is embedded in societally and culturally ingrained misogyny.^17^ Historically, the perception of beauty has been used to oppress women, whereby maintenance of fertility and aesthetics was of paramount importance. The use of beauty as a tool of oppression has become known as the “beauty myth” and was analyzed by feminist writer Naomi Wolf in her book “The Beauty Myth: how images of beauty are used against women.”^20^ This myth is evident in the medical literature today, highlighted by recent mainstream media controversies over doctors rating the aesthetic attractiveness of their endometriosis patients.^2,21^ Wolf asserts that the “beauty myth” is a tool of oppression and a political weapon against women.^20^

The “hidden curriculum” may be an important contributor to the outcomes of the androcentricity of medical research on the experiences of female patients. The hidden curriculum pertains to the aspects of medicine that are not formally taught to medical students: the attitudes and values collected from their experiences with senior clinicians on the wards and in general practice. Perceptions from androcentric medical research, whereby female patients do not fit the male mould and, therefore, are “difficult” or “mad,” may be passed down through the hidden curriculum to junior staff members, thereby continuing the cycle.^22^ In addition, medical students arguably become acculturated to the historically masculine medical environment that demands conformity.^18^

As more is learnt about the gender gap and misogyny in medical research, and the impact this has upon female patients, it is important to determine whether misogyny continues to permeate the narrative in modern medicine. It is also important, while examining misogyny in medical research, to realize that sex and gender are distinct, yet the terms are often used interchangeably. Sex refers to the biological and physiological characteristics that define humans as male, female, or intersex.

Gender, rather, is a societal construct that refers to roles, activities, and behaviors, and encompasses a wide range of identities beyond male, female, and intersex.^23^ In this systematic scoping review, we aim to explore the extent of study into sex and gender gaps in the published literature and assess whether misogynistic characterization is prevalent in contemporary medical research.

## Methods

### Search strategies and study selection

A three-phase approach to searching the literature was employed. Initial literature searches were conducted by one author (L.M.) using PubMed, Science Direct (Elsevier) PsychINFO, and Google Scholar, in line with current recommendations (9). Key words were used for each database and combined with Boolean operators AND and OR. A second search of each database was performed using different terms. All search terms are outlined in Table 1. Finally, a citation search was performed to identify studies that may not have been captured in the search terms.

**Table 1.** Search Terms

Search number	PubMed (MeSH)	Science Direct	PsychINFO	Google Scholar
1	Sex	Sex	Sex	Sex
	Translational medical research	Gender	Gender	Gender
	Biomedical research	Sexism	Sexism	Sexism
	Gender gap	Research	Research	Research
		Gender gap	Gender gap	Gender gap
		Medical research	Medical research	Medical research
2	Women	Women	Women	Women
	Aesthetic	Sexism	Sexism	Sexism
	Disease	Attractive	Attractive	Attractive
	Research	Aesthetic	Aesthetic	Aesthetic
	Attractive	Research	Research	Research

### Inclusion criteria

Selected articles included all peer-reviewed journal publications published between January 01, 2009, and January 01, 2019. Studies were restricted to English language articles, and those where full text was not available were excluded. Opinion pieces, editorials, and nonpeer-reviewed publications were also excluded. An article was deemed to display misogyny if it discussed/assessed the female aesthetic in terms of health, but either did not measure health, or if the research could not be deemed useful or beneficial for clinical practice.

A total of 30 full texts were assessed by two authors using stringent inclusion and exclusion criteria (Table 2); a further 13 were excluded, of these 6 were editorials or reports rather than research, 2 were based on the gender gap in scientists rather than patients, and the remaining 5 examined clinical care and interventions rather than the presence of a gender gap in the literature. A total of 17 articles were included in the qualitative analysis.

**Table 2.** Inclusion and Exclusion Criteria

Inclusion criteria	Exclusion criteria
Research article	Opinion piece, editorial, etc.
English and available	Non-English or unavailable
Examines the gender gap in medical research or focuses on female aesthetics in medicine	Examines the impact of gender on clinical care
2009–2019	Before 2009

### Data extraction

The results were merged and duplicates removed using Endnote X9.3. Data were collected from the 17 included articles using a data extraction tool (Table2) to collect the study name, lead author and year, methods, aims and objectives, and results. Aims and objectives were taken as direct quotes from the articles where possible, to avoid any interpretation bias. Analysis was conducted according to the 2009 Ppreferred Rreporting Iitems for Ssystematic Rreviews and Mmeta-Aanalysis (PRISMA) checklist.^24^

**FIG. 1.**
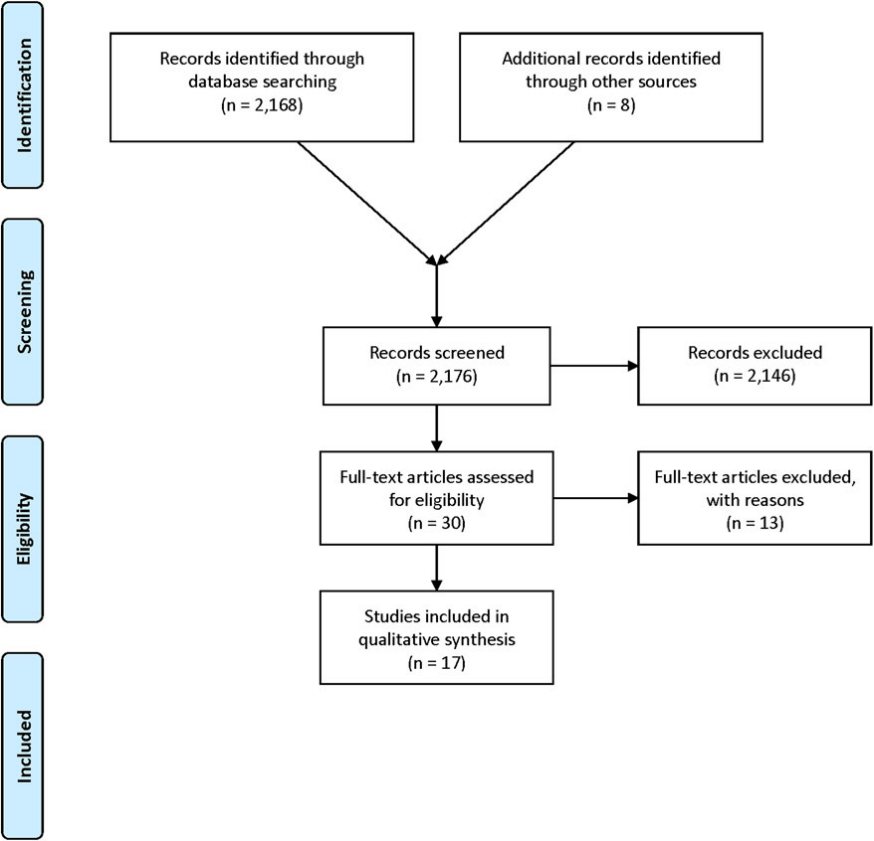
PRISMA flow chart of the search strategy. PRISMA, Ppreferred Rreporting Iitems for Ssystematic Rreviews and Mmeta-Aanalysis.

As demonstrated in the PRISMA flow chart (Fig. 1), 2176 articles were identified by the study search, 2146 were excluded due to lack of relevance. Thirty full texts were assessed for eligibility, with 13 excluded as they did not meet the inclusion criteria. Seventeen articles were included in the analysis.

## Results

Of the 17 included articles, 12 examined the gender gap in medical research and 5 demonstrated misogyny, assessing female attractiveness for alleged medical reasons. Three studies were reviews of the published literature^25–27^ defined as collation of all empirical evidence that fits prespecified eligibility criteria,^28^ nine were cross-sectional analyses^29–38^ (observational studies of the published literature over a defined time period^39^), and the five remaining studies were within-subject experimental design.^40–43^ Of the 12 studies examining gender gaps (Table 3), 11 were conducted in the United States^25,26,29–33,35–38^ and 1 in Canada.^34^

**Table 3.** Results: Representation of Female Participants in the Contemporary Medical Literature

Study name, authors, year	Methods/description	Specialty	Study aims/objectives	Results
“Representation of women in randomised controlled trials of cardiovascular disease prevention” Melloni et al. (2010)^25^	Reviewed the literature for female representation. Examined 156 clinical trials of CVD prevention	Cardiology	“To determine female representation over time and by clinical representation”	135 (86.5%) of the trials examined recruited both male and female participants, 20 recruited just males, and 1 recruited just females. Proportion of women in the trials increased from 9% in 1970 to 41% in 2006. Enrollment of women in RCTs for CVD has increased but remains low relative to population disease prevalence. Female representation was high for trials for the diseases: hypertension, diabetes, and stroke, and lowest for heart failure, coronary disease, and lipidemias, Females were represented in 30% of trials used to produce heart disease prevention guidelines.
“Inclusion, analysis and reporting of sex and race/ethnicity in clinical trials: have we made progress?” Geller et al. (2011)^29^	Cross-sectional analysis of the published literature. Identified RCTs in 9 journals through 2009, 86 were eligible for analysis.	Nil specific	“To determine the current level of compliance with these guidelines for the inclusion, analysis, and reporting of sex and race/ethnicity in federally funded randomized controlled trials and to compare the current level of compliance with that from 2004”	In total, 30/86 were sex specific. Of those that were not, the median enrollment of women was 37%. Seventy-five percent did not report outcomes by sex. Nine studies had fewer than 20% female participants. Only three studies noted their lack of diversity as a limitation.
“Participation of women and sex analyses in late-phase clinical trials of new molecular entity drugs and biologics approved by the FDA in 2007–2009.” Poon et al. (2013)^30^	Cross-sectional analysis of clinical trials. Evaluated LPCTs and BLAs for women's participation, 2007–2009.	Nil specific	To provide “an update on the current status of the participation of women in late-phase clinical trials (LPCTs) submitted to support the approval of new drugs and biologics by the FDA”	Female participation in LPCTs was 43% (decrease from 52% in 2001) and 57% (increase from 45% 1995–1999) for BLAs.
“Sex bias exists in basic science and translational surgical research” Yoon et al. (2014)^26^	Authors reviewed 2347 articles for sex reporting	General surgery	To determine “that sex bias exists in surgical research”	Sex reporting was poor; for animal studies 22% did not specify sex, and where sex was specified, 84% used male animals only. For cell research, 76% did not report sex, again where sex was reported, 71% were male. For female-prevalent diseases, 44% did not specify sex studied and those that did, only 12% used female animals.
“Determining if sex bias exists in human clinical research” Mansukhani et al. (2016)^31^	Cross-sectional analysis of published literature. Extracted data from 1303 research articles across five clinical journals	Nil specific	“To determine if sex bias exists in human surgical clinical research”	In total, 38.1% of studies reported data by sex, 33.2% analyzed results by sex, and 22.9% discussed implications of sex in the results. Few studies included men and women equally. Inclusion and matching of the sexes varied greatly.
“Mind the gap: sex bias in basic skin research” Kong et al. (2016)^33^	Evaluated skin research publications between 2012 and 2014 to determine whether the sex was disclosed	Dermatology	To “explore how often discovery in cutaneous biology stems from the study of one sex”	No sex information was provided in 60% of the studies of cells from humans and animals. Where sex was declared, 70% were male.
“Reporting of sex and gender in randomised controlled trials in Canada: a cross-sectional methods study” Welch et al. (2017)^34^	Cross-sectional examination of gender reporting and proportions in the random cross section of 100 articles published in the Canadian medical literature	Nil specific	“Provide preliminary assessment of the extent and nature of reporting about sex and/or gender, including whether sex/gender analysis was carried out”	No study analyzed the effect of sex/gender on their results, and when they were considered, it was focused on the biomedical differences alone, despite the fact >50% of studies examined nonpharmacological interventions. Only 6% performed gender/sex subgroup analysis. No article defined sex/gender. No RCT mentioned gender-diverse populations.
“Participation of women in clinical trials supporting FDA approval of cardiovascular drugs” Scott et al. (2018)^32^	Cross-sectional analysis of published literature. Assessed enrollment of women in 36 drug trials from 2005 to 2015.	Cardiology	“To examine women's participation and the reported safety and efficacy by gender for pivotal cardiovascular disease (CVD) trials submitted to the U.S. Food and Drug Administration (FDA) supporting marketing applications”	Proportion of women enrolled ranged 22–81% with a mean of 46%. Women were well represented in studies examining hypertension and atrial fibrillation, over-represented for studies exploring pulmonary hypertension and under-represented in heart failure and coronary artery disease studies.
“Sex bias in hand surgery research” Kalliainen et al. (2018)^35^	Cross-sectional evaluation of the recent literature for inclusion of both sexes in research and the use of sex as a dependent variable in hand surgery. In total, 386 studies published in 4 journals for 2 years.	Hand surgery	“To provide a jumping-off point for the conversation in the hand-surgery community about sex-based outcomes”	An equal number of males and females were included in clinical studies; however, many individual studies did not include equal numbers of each gender. The female–male ratio depended on the pathology and surgery required.
“The more things change, the more they stay the same: a Study to evaluate compliance with inclusion and assessment of women and minorities in randomised controlled trials” Geller et al. (2018)^36^	Cross-sectional analysis of the published literature. Examined 782 RCTs across 14 journals in 2015	Nil specific	“To investigate current levels of compliance with guidelines for inclusion, analysis and reporting in NIH-funded RCTs and compare the results with those from 2009 and 2004”	In total, 35 studies enrolled just one sex, median enrollment of women in the remaining studies was 46%, however, 15% of the studies enrolled fewer than 30% women. Only 26% reported the effect of sex as a covariate. The NIH guidelines from the NIH revitalization act 1993 have not resulted in significant increases in reporting results by sex.
“Count me in: using a patient portal to minimise implicit bias in clinical research recruitment” Kannan et al. (2019)^37^	Cross-sectional study offering patients who utilize an online patient portal the opportunity to volunteer for the research recruitment registry (U.S.).	Nil specific	“Determine differences in volunteering to join a research recruitment registry between men and women”	Women volunteered for the research recruitment registry at a slightly greater proportion than that of all portal users. Supports theories that bias against women in clinical recruitment is due to bias rather than women's unwillingness to be involved.
“Factors affecting sex-reporting in medical research; a cross-sectional bibliometric analysis” Sugimoto et al. (2019)^38^	Bibliometric cross-sectional analyses of >1.5 million articles published during 1980–2016 were examined for sex reporting.	Nil specific	To determine the “degree of sex-reporting across health sciences and the role of gender in sex-related reporting.”	Sex-related reporting increased over the years. Articles with female lead authors had greater chance of reporting sex (OR 1.26). In biomedical research, sex remains under-reported (31%). Scarcity of women in science may be related to inadequate reporting of sex in research.

BLA, biological license application; CVD, cardiovascular disease; LPCT, late phase clinical trial; RCT, randomized controlled trial.

The five experimental design studies were the same five studies that demonstrate misogyny (Table 4). These five studies were conducted in the United Kingdom (*n* = 2),^40,41^ Poland (*n* = 2),^42,43^ and Italy (*n* = 1).^21^ Results are presented in Tables 3 and 4.

**Table 4.** Results: Evidence of Misogyny in the Contemporary Medical Literature

Study name, authors, year	Methods/description	Specialty	Study aims/objectives	Results
“Judging the health and attractiveness of female faces: is the most attractive level of facial adiposity also considered the healthiest?” Coetzee et al. (2011).^40^	Males and females from a university cohort were asked to transform the adiposity in photographs of female's faces to optimize attractiveness and then optimize health.	Nil specific	“To test if people differentiate between the level of facial adiposity they find attractive and healthy in female faces”	Females determined there is a significant difference between a healthy level of adiposity and an attractive level of adiposity. For males the difference was far smaller.
“Attractiveness of women with rectovaginal endometriosis: a case-control study” Vercellini et al. (2013)	Four physicians (two male, two female) were asked to evaluate the attractiveness of women undergoing surgery for endometriosis on a likert scale. Physical examination of the women included measurement of the breast–underbreast ratio.	Gynecology	“To evaluate the physical attractiveness of women with and without endometriosis”	Women with rectovaginal endometriosis were deemed by researchers to be more attractive than those with endometriosis of other locations. These women were also leaner, had larger breasts, and earlier coitarche.
“Female facial appearance and health” Gray et al. (2012)	In total, 105 female participants were photographed and then undertook a self-reported health questionnaire regarding bouts of rhinovirus or influenza. Observers rated the photographs for femininity, attractiveness, health, makeup and mood.	Nil specific	To determine whether femininity, health, and attractiveness would “correlate negatively with bouts of illness”	There was a general trend for facial femininity and attractiveness to correlate negatively with bouts of upper respiratory tract illness, but not with gastrointestinal illness. Concluded that facial attractiveness and femininity may indicate a woman's health history.
“Costs of reproduction are reflected in women's faces: post-menopausal women with fewer children are perceived as more attractive, healthier and younger than women with more children” Marcinkowska et al. (2018)	In total, 571 male and female participants evaluated photographs of the faces of 30 women of varying parity and asked to choose the faces they found most attractive, younger, and healthier.	Nil specific	To determine whether the “high costs of reproduction” are “perceived by others when they evaluate facial attractiveness”	Women who had given birth to fewer children were judged as more attractive, younger, and healthier by both male and female participants. Examination of historic photos determined that more attractive younger women had higher reproductive success.
“Analysis of the visual perception of female breast aesthetics and symmetry: an eye tracking study.” Pietruski et al. (2019)^43^	Eye-tracking technology was utilized for 100 participants (50 male, 50 female) observing images of different female breasts. Attractiveness was determined by duration and location of gaze fixation.	Breast surgery	To “objectively analyse the visual processes taking place during the assessment of female breast aesthetics and symmetry” for “more reliable surgical outcomes”	Key characteristics of gave patterns for males and females were the same. Most fixations were on the nipple/areola area, suggesting it is a key area for symmetry. More attention was paid to lower breast shape than upper breast/chest/clavicle areas. Authors state it is unknown how visual patterns translate to assessment of attractiveness.

Three themes were identified: females remain under-represented in biomedical research, sex and gender are poorly analyzed and reported in research, and several contemporary research articles display ideas that can be construed as misogynistic.

### Proportion of females versus males in clinical trials

A total of seven published articles analyzed the proportion of females–males participating in research trials.^25,30–32,34–36^ Under-representation of women was noted, largely because of discrepancies between specialities, with some specialist fields recruiting more female participants than male participants, and others recruiting fewer females than males. Kong et al. observed that in cell biology, 60% of studies gave no information on the sex of the cells studied.^33^ Two studies noted that specifically cardiovascular research appeared to under-represent women.^25,32^

### Reporting and analysis by sex and gender in clinical trials

Six published articles examined the analysis and reporting of results by sex or gender.^26,29,31,33,34,38^ These articles found that analysis by sex or gender was somewhere in the range of 6%–38%. Sugimoto et al. noted that articles with authors' with female names were more likely to report results by sex or gender.^38^

### Examples of misogyny in medical research

Five articles displayed ideas that could be perceived as misogynistic^21,40–43^ with three of these five studies stating that they assess female attractiveness as a marker of health.^21,40,42^ Pietruski et al. conducted an analysis of visual tracking of the aesthetic of breasts, however, also stated their method is potentially flawed in that there is limited evidence as to how visual patterns translate to assessment of attractiveness.^43^

## Discussion

This review highlights several issues regarding sex and gender in the medical literature. Females remain broadly under-represented in the medical literature, sex and gender are poorly reported and inadequately analyzed in research, and misogynistic articles continue to permeate the narrative.

### Females are largely under-represented in biomedical research

Several studies determined that women are largely under-represented in medical research. Geller et al. analyzed 86 randomized controlled trials (RCTs) across 9 journals and found female representation to be just 37%, with only 3 studies noting the limitations of lack of diversity.^29^

Perception of the disease, rather than actual sex prevalence, appears to drive the representation of females in medical research.^44^ For diseases that are perceived to affect men more than women, androcentricity dominates the research picture. Melloni et al. examined the representation of women in cardiovascular disease prevention RCTs. Although most studies in Melloni's analysis recruited both sexes and the proportion of women participants was noted to be increasing, there were still 20 studies noted to have recruited only male participants, versus just 1 study with only female participants recruited. Importantly, female representation was higher in diseases perceived to affect women more, such as hypertension and stroke.^25^

Similarly, Scott et al. also examined the participation of women in cardiovascular drug trials, and determined that in diseases believed to affect women in greater numbers than men, such as hypertension, atrial fibrillation, and pulmonary hypertension, women were either adequately represented or over-represented. In diseases perceived to affect greater number of men than women, such as coronary disease and heart failure, females were under-represented.^32^ Kalliainen et al. noted that the female–male recruitment ratio in hand surgery research was dependent on the pathology, but the ratios recruited were roughly in keeping with sex prevalence of the disease,^35^ noting a need to increase members of the less-represented sex to enhance statistical power.

The *1993 National Institutes of Health Revitalisation Act* recommends that women and men be included in clinical trials based on the sex prevalence of the disease and to provide data on the efficacy of treatment in each sex,^45^ which may help account for the under- and over-representation of females depending on disease. However, the perceptions of sex-related prevalence appear frequently to be outdated; for example, coronary artery disease is the commonest cause of death in both men and women^46^ and women experience greater functional disability and symptoms burden and a higher prevalence of nonobstructive coronary artery disease than men.^47^

Similarly, women make up 30% of the gout disease population; however, they make up just 5.3% of gout clinical drug trial participants.^30^ Geller et al. conducted a cross-sectional study of RCTs across 14 journals published in 2015 and determined that the guidelines from the *1993 National Institutes of Health Revitalisation Act* have not resulted in significant increases in reporting results by sex.^36^ Indeed, Poon et al. noted in a similar study that female participation in clinical trials has decreased since the 1990s.^30^

Not only is it important to acknowledge outdated gender-based beliefs, the representation and participation of women in medical research are important because medical research informs the development of clinical guidelines. Clinical guidelines directly impact the lives of patients, therefore, if there are sex and gender differences in presentation, management, and clinical response to management, it is vital these are described. Melloni et al. conducted an analysis of published RCTs and determined that in clinical trials used to inform guidelines for cardiovascular disease prevention in women, female participation was just 30%.^25^

Kannan et al. explored recruitment bias using a cross-sectional study of patients volunteering for research through an online portal and concluded that under-representation of women in clinical trials is not due to the unwillingness of women to volunteer, but rather owing to bias within trial design and recruitment.^37^

### Sex and gender are poorly reported and analyzed in contemporary biomedical research

Lack of female representation in research was noted consistently throughout review of the literature, however, another related issue was the lack of sex reporting or inclusion in analysis of many medical and biomedical studies. Kong et al. evaluated sex bias in published dermatological research, much of which is on nonhumans such as cell lines or animals; 60% of the cellular studies from both humans and animals provided no information regarding sex. Where sex was declared, 70% of the cell lines studied were from males.^33^

Welch et al. supported this observation in a cross-sectional study of RCTs in Canada, finding that no studies considered the influence of sex and only 6% of studies performed a subgroup analysis for sex.^34^ Similar findings are noted consistently in other studies, and sex reporting appears particularly poor in animal and cellular studies.^26,31,38^ Analyses of results by sex are seemingly poor across all study types.^26,31,34^

Some of the gender discrepancy has been explained by Hankivsky et al.; examination of statements surrounding sex and gender inclusion from 45 health-research funding agencies and 10 sex/gender health journals determined there is little consistency in whether sex and gender are mentioned in funding and publication guidelines. There is also significant variation in the conceptualization of sex and gender and how researchers address this in research. The criteria set by agencies fail to address the complex relationship between sex and gender and health.^27^

#### Misogyny is evident in the medical research

A total of five studies exhibited misogyny. Vercellini et al. utilized a likert scale for clinicians to subjectively assess the attractiveness of women undergoing surgery for endometriosis.^21^ Although this publication caused outrage in the global media and across social platforms such as Facebook and Twitter,^2^ it is far from unique. Three studies proposed to assess the attractiveness of the female face as a measure of health, however, none of them utilized objective measures of health.^40–42^

Marcinkowska et al. determined that women with higher parity are perceived as less attractive and healthy, but the study did not provide information regarding objective measures of health from which this conclusion was generated.^42^ A further study by Pietruski et al. applied eye-tracking technology to ascertain the attractiveness of female breasts in the context of reconstructive surgery; however, authors acknowledged that the link between lingering gaze and attractiveness is unknown,^43^ raising the question of why this study was conducted.

Studies displaying misogyny are problematic for clinical medicine and patient care for several reasons. First, they continue to perpetuate the concept of the “beauty myth” and the utilization of the female aesthetic as a tool of oppression and prejudice. In highlighting female beauty, the authors of the articles inadvertently suggest that patient care should be altered based on the aesthetic of the patient. Despite the findings of Vercellini et al. that attractive women are more likely to have severe endometriosis,^21^ it is unlikely that this will be incorporated into clinical practice given the subjective nature of the assessment.

Consequentially, studies that focus on the female aesthetic add little to the care of women and may be deemed unethical. Furthermore, the beauty myth has been debunked, evidenced by the evolving nature of society's preference of female aesthetics, from the voluptuous women of the 1940s and 1950s to the emaciated females of the 1970s to 1990s, and finally to the contemporary fashion of fitness and athleticism as beautiful.

As Wolf writes, “there is no legitimate justification for the beauty myth,” but rather the emphasis on beauty is about power and patriarchy, where the aging woman is most feared as advancing age is associated with increased knowledge, power, and respect.^20^ Possibly as a result of the beauty myth, women's identities may be entrenched in their physical attractiveness, which ensures their vulnerability to external approval.^20^

Failure to achieve the gold standards to beauty impacts on self-esteem and possibly subsequent success.^2,48,49^ Studies focusing on female attractiveness may serve to reinforce false beliefs surrounding beauty and increase hostility toward women.^50^ Hostile sexism may lead to perpetuation of the hysterical discourse^51^ and increase time to diagnosis and occurrence of misdiagnoses.

Although sexism remains present in the medical sphere, patient care is compromised. The research gap and the publication of misogynistic research may adversely affect female care and contribute to the negative perceptions of female hysteria and the gap in time to diagnosis experienced by many women. The androcentric history of medical research led to assumptions about women's health and response to treatments based solely on studies from male bodies.^10,17^ A rapidly growing body of evidence from clinical research demonstrates that females and males can differ greatly in their susceptibility and presentation of disease and their response to treatment and profile of adverse effects.^23^

A more immediate issue with studies that display misogyny is the trust patients place in the medical profession. Women reportedly feel a sense of distrust in the medical profession, with many professing acute awareness that doctors have the power to label them as “anxious” or “depressed” rather than address their physical pain or symptoms.^17^

Physicians assessing physical attractiveness as part of a clinical assessment is arguably entering dangerous territory when it comes to the doctor–patient relationship.^2^ Doctors are bound by the Hippocratic Oath and a set of laws from registering boards, under which the boundary of relations with patients is clear and absolute.^52^ Rating patient attractiveness is, therefore, both inappropriate and extremely subjective, consequentially it must not be allowed to blur the clinical picture.

Other countries have developed research policies to address the sex and gender imbalance in research and to stipulate that sex must be reported; Australia has somewhat lagged on this.^1,23^ Until we address the gender gap and misogyny in medical research, we run the risk of the health care of female patients remaining substandard to the care of male patients.^53^ The consequences of neglecting sex and gender differences are wide reaching—including inaccuracies in science, adverse health outcomes, and experiences and cost ineffectiveness.^23,54^

### Limitations

This review has a few limitations, first in that sex and gender are largely considered in binary terms and there is no discussion of intersexuality, nonbinary, transgender, or any others on the gender spectrum because there is very limited literature on gender gaps in these population groups, and the authors highlight a need for further research in this area. Second, only articles from the recent decade were included, this was to allow the review to examine only the most contemporary literature, however, it is likely that more historical review would yield more evidence of misogyny and gaps in gender analysis.

Finally, the definition of misogyny in research was determined by the authors, because there was no literature previously examining this topic in the medical literature, there is no gold-standard way to measure the misogyny of a research article. In much the same way as the Bechdel test for positive female representation within media,^55^ the authors propose it may be of use to develop a tool to assess misogyny in medical research and to provide guidelines to avoid future publication of misogynistic research.

## Conclusions

The gender gap and misogynistic studies, which serve little to improve women's health, remain present in the contemporary medical literature. There may be several drivers for the gender gaps and misogyny revealed by this scoping review, including outdated perceptions of sex prevalence of diseases, unhelpful discourse surrounding female patients and illness, and the ongoing perpetuation of the beauty myth. The hidden curriculum in medical education may be an important and seemingly unexplored contributor to the outcomes of the androcentricity of medical research on the experiences of female patients.

## Abbreviations Used

BLA: biological license application
CVD: cardiovascular disease
FDA: Food and Drug Administration
LPCT: late phase clinical trial
MI: myocardial infarction
PRISMA: preferred reporting items for systematic reviews and meta-analysis
RCT: randomized controlled trial
